# GATR: A Road Network Traffic Violation Prediction Method Based on Graph Attention Network

**DOI:** 10.3390/ijerph20043432

**Published:** 2023-02-15

**Authors:** Yuquan Zhou, Yingzhi Wang, Feng Zhang, Hongye Zhou, Keran Sun, Yuhan Yu

**Affiliations:** 1 School of Earth Sciences, Zhejiang University, Hangzhou 310058, China; 2 Department of Traffic Management Engineering, Zhejiang Police College, Hangzhou 310053, China; 3 Zhejiang Provincial Key Laboratory of Geographic Information Science, Hangzhou 310058, China

**Keywords:** traffic violation, spatiotemporal prediction, graph attention network, road network, urban function

## Abstract

Prediction of traffic violations plays a key role in transportation safety. Combining with deep learning to predict traffic violations has become a new development trend. However, existing methods are based on regular spatial grids which leads to a fuzzy spatial expression and ignores the strong correlation between traffic violations and road network. A spatial topological graph can express the spatiotemporal correlation more accurately and then improve the accuracy of traffic violation prediction. Therefore, we propose a GATR (graph attention network based on road network) model to predict the spatiotemporal distribution of traffic violations, which adopts a graph attention network model combined with historical traffic violation features, external environmental features, and urban functional features. Experiments show that the GATR model can express the spatiotemporal distribution pattern of traffic violations more clearly and has higher prediction accuracy (RMSE = 1.7078) than Conv-LSTM (RMSE = 1.9180). The verification of the GATR model based on GNN Explainer shows the subgraph of the road network and the influence degree of features, which proves GATR is reasonable. GATR can provide an important reference for prevention and control of traffic violations and improve traffic safety.

## 1. Introduction

As reported by the World Health Organization (WHO), 1.3 million people die in road traffic accidents every year around the world, which is the top killer of children and young people aged 5 to 29 [1]. Traffic violations are the behavior of individuals related to traffic activities who violate relevant traffic management laws and regulations. Typical traffic violations include drunk driving, unlicensed driving, overloading, reverse driving, not wearing seat belts, etc. Traffic violations seriously threaten the traffic order and road safety, and also cause huge economic losses and casualties. Traffic violation has become the most important risk to road safety [2,3]. The distribution of traffic violations has significant spatiotemporal heterogeneity [4,5] affected by various factors such as people, vehicles, roads, and the environment [5,6,7,8,9]. Traffic violation features refer to the factors related to the occurrence of traffic violations and are always used to predict traffic violations. Traffic violation features can be divided into two categories: internal features and external features. Internal features refer to the factors directly involved in traffic behaviors, including drivers and vehicles; external features refer to the factors that have an implicit impact on the traffic system, including road conditions, weather, temperature, date, etc. Considering the features and predicting the spatiotemporal distribution of traffic violations are of great significance for taking prevention and control measures in advance.

The prediction methods of traffic violations mainly include traditional statistical methods and deep learning methods. Most of the early research on traffic violation prediction used statistical methods including clustering, regression, frequent tree and non-negative matrix decomposition [10]. These statistical methods establish traffic violation prediction models by quantifying the risk variables in the traffic system. Anderson et al. (2009) used the kernel density estimation method to analyze the traffic violations in London [11]. They extracted the hot spots of traffic violations, divided the basic spatial units according to the density, used the K-means method to cluster the basic spatial units combined with the environmental features, and finally identified 15 different risk spatial areas. Caliendo et al. (2007) used Poisson distribution, negative binomial distribution and negative polynomial distribution to predict traffic violations [12]. Lin et al. (2015) proposed a traffic violation prediction method based on the frequency tree. They first identified all frequent patterns in the traffic violation data set, estimated the importance of each variable in the frequent patterns through the value of ROPR (relative object purity ratio), and then filtered out the variables that contribute most to the pattern. Finally, the traffic violations were predicted using specific variables based on a Bayesian network model [13]. Chen et al. (2018) selected road network data, POI (point of information) data and population density data as characteristic matrices, used a non-negative matrix decomposition method to decompose the matrices into low-rank matrices, and then shared them to transfer information and similarity, so as to improve the accuracy of traffic violations prediction [14]. Yue et al. (2018) adopted the multiple time series classification method to extract three novel features to measure the severity of traffic violation. The method took into account the inherent time pattern in the traffic data, resulting in high prediction accuracy [15]. However, the statistical methods ignore the spatiotemporal dependence of traffic violations, resulting in poor prediction accuracy and no spatial distribution.

In recent years, deep learning methods have been widely adopted. These methods used traffic violation features to predict the number and spatial distribution. Zeng et al. (2017) proposed a new soft attention RNN (recurrent neural network), which models the nonlinear interaction in space and appearance between static regions, and realizes advanced prediction [16]. Chan et at. (2016) proposed a dynamic attention RNN to predict accidents in the video of the tachograph based on the appearance and motion characteristics of objects [17]. In order to consider the significant spatiotemporal characteristics of traffic violations, CNN (convolutional neural network) and LSTM (long short-term memory artificial neural network) are commonly used [18]. Najjar et al. (2017) collected satellite image data to predict traffic violations based on a CNN. The accuracy of the prediction reached 78% in New York City [19]. The CNN took the surrounding area of the target area into consideration through convolution, which partly satisfied the spatial relevance of traffic violations. Ren et al. (2017) collected a large number of heterogeneous relevant data to predict traffic violations based on LSTM [20]. The accuracy indicators of the LSTM model are better than LR (logical regression), DT (decision tree), SVM (support vector machine), etc. The LSTM model can identify and capture the time correlation of traffic violations [21]. However, traffic violations have both spatial and temporal correlation, and modeling on a single dimension cannot fully express the characteristics of traffic violations. Therefore, whether the CNN model based on spatial dimension or the LSTM model based on time dimension is used, the accuracy is not ideal enough. Yuan et al. (2018) selected the internal and external features of the traffic system such as weather, road conditions and traffic volume as the data set, comprehensively applied CNN and LSTM methods, and proposed the Conv-LSTM method to predict traffic violations [22]. The prediction accuracy of Conv-LSTM in Iowa City is better than the CNN, LSTM and other deep learning methods [23]. Conv-LSTM took into account the advantages of both CNN and LSTM, captured the correlation of traffic violations from both dimensions of time and space, and achieved higher prediction accuracy.

Although the existing traffic violation prediction methods have achieved good prediction accuracy, the following problems are still prominent: (1) Most existing methods divide the study area into regular spatial grids and map traffic violations onto the grids, while regular spatial grids completely ignore the important role of roads. In other words, the regular space grids can only locate traffic violations onto a specific space range, which is meaningless except for its location. Traffic violations always occur on the road, thus strongly relate to the road network and affect each other along the road network. The restriction of the road network can help better express the spatial characteristics of traffic violations. The road network should not be ignored in traffic violation prediction. (2) Traffic violations are the result of various internal and external features. Social features are considered to be one of the most import external features in the traffic violation. However, most existing methods do not take them into account. For example, different urban areas carry different functions, which has an important effect on the resident behavior, and different residents at specific times and functional areas will produce different traffic flows, which has a great impact on the occurrence of traffic violations. So, the urban function and other social features should be fully considered in the prediction model.

To solve the above problems, we propose the GATR (graph attention network based on road network) model to predict the spatiotemporal distribution of traffic violations. The road network has strong self and mutual correlation [24]. GATR adopts a topological graph and attention mechanism which can well express the spatiotemporal dependency [25]. On the one hand, GATR adopts the topological graph as the research object. The nodes of the graph are abstracted from every section of the road network, which can accurately locate traffic violations on the road sections. The edges represent the connectivity of two nodes, and thus represent the connectivity of the road segments. Furthermore, GATR adopts the attention mechanism to adaptively adjust the weight of each road segments, which can more accurately calculate the impact degree of other nodes on the target node. Compared with the regular spatial grids used by existing methods, the topological graph and attention mechanism used by GATR help it better consider the spatiotemporal correlation of traffic violations along the road network. On the other hand, traffic violations are greatly affected by road type, the number of lanes, speed limit, volume etc. [26,27,28] We construct a traffic violation feature set to predict its spatiotemporal distribution. The traffic violation feature set contains historical traffic violation features, external environmental features and urban functional features. All the features have significant impact on the occurrence of traffic violations.

Compared with Conv-LSTM, the GATR model has significant advantages in prediction accuracy and spatiotemporal accuracy. In addition, we use GNN Explainer to verify the GATR model, showing the road influence subgraph of the basic road units and the influence degree of different features on traffic violations, which indicate the GATR model is reasonable. Predicting traffic violations through the GATR model has significant reference value for taking prevention and control measures in advance.

## 2. Materials

### 2.1. Study Area

**Figure 1:**
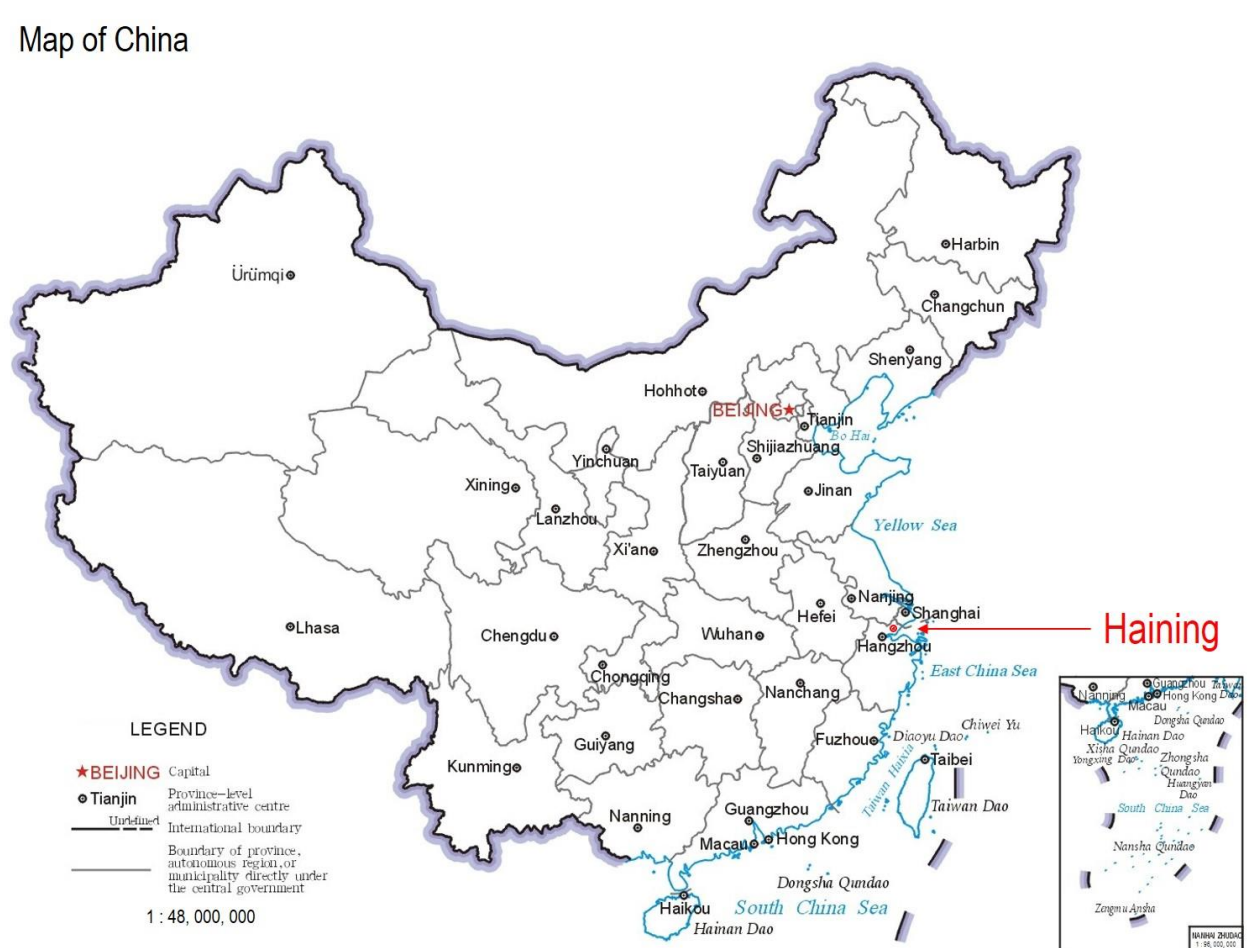
The location of City H in China. The base map is supervised and produced by the Ministry of Natural Resources of the People’s Republic of China, and the review number is GS(2019)1673.

The study area is City H, whose full name is “Haining” located in Jiaxing, Zhejiang Province, China. The location of City H in China is shown in Figure 1. City H is located on the Hangjiahu Plain, on the north bank of the Qiantang River. It has a subtropical monsoon climate with four distinct seasons, one of which is both rainy and hot. City H has a total area of 863 square kilometers, with 4 streets and 8 towns under its jurisdiction, and a population of 1099.4 thousand. City H is close to Hangzhou, Shanghai, and enjoys a prosperous economy with a regional GDP of CNY102.078 billion. It is also one of the top 100 counties in China.

**Figure 2:**
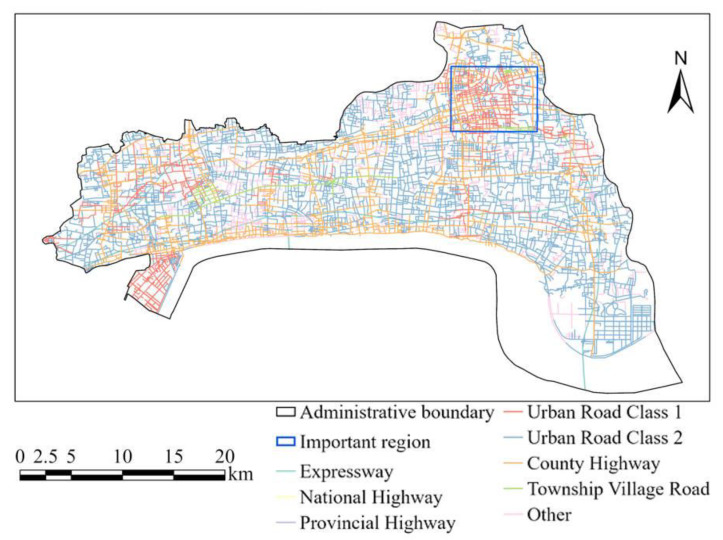
City H road map including expressway, national highway, provincial highway, urban road class 1, urban road class 2, county highway, township village road and others.

City H has a well-developed transportation network with a length of 1694.0 km, including 7.5 km of national roads, 59.7 km of provincial roads and 328.5 km of county roads. It relies on the two horizontal (Shanghai Hangzhou Expressway and Hangzhou Pujiang Expressway) and three vertical (Hangzhou belt, Xiaoshan Haining River crossing, and Suzhou Jiaxing Shaoxing Expressway) expressway network externally, and has built a regional trunk road network of one ring, three horizontal, ten vertical and ten links internally [29]. Road distribution of City H is as shown in Figure 2. The blue rectangle covers the high incidence area of traffic violations.

### 2.2. Datasets

The datasets include the City H road network, traffic violation features, POI dataset and traffic violations. The road network dataset comes from OpenStreetMap (https://www.openhistoricalmap.org/, accessed on 1 June 2022) including main traffic arteries such as expressways, national highways, provincial highways, etc. The traffic violation feature dataset includes historical traffic violation features, external environmental features and urban functional features. The historical meteorological dataset comes from the weather network (http://lishi.tianqi.com/, accessed on 1 June 2022). The historical date dataset comes from the perpetual calendar website (https://wannianrili.bmcx.com/, accessed on 1 June 2022). POI is the ground location attached various information which can help to identify the urban function. The POI dataset comes from Gaode open platform (https://lbs.amap.com/, accessed on 1 June 2022), the types of which are divided into six categories: residential land, public management and public service facilities land, commercial service facilities land, industrial land, road and traffic facilities land, and green space and square land. The dataset of traffic violations comes from the public security organ, and the time range is from 1 January 2016 to 31 December 2020. The dataset has been strictly desensitized and does not contain personal information and other privacy-sensitive contents.

## 3. Methods

### 3.1. GAT

GNN is a neural network model based on graph data structure. GNN establishes a topological graph by nodes and edges. The nodes are abstracted from research objects and the edges are the relationship of them. CNN can only handle the matrix, which is regular data, while GNN has stronger expression ability for irregularly distributed data [30].

**Figure 3:**
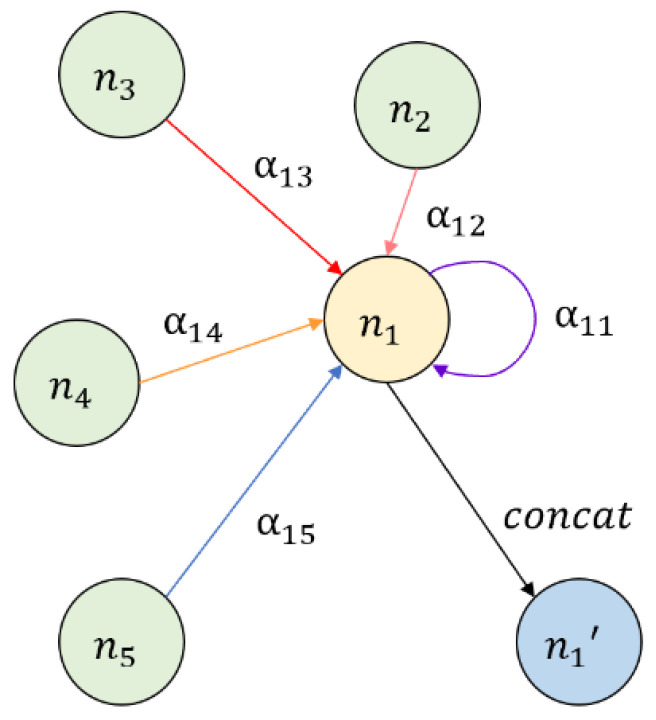
Attention mechanism of GAT.

CNN adopts regular spatial grids as the research object and uses convolution to consider the influence of adjacent grids around the central grid. The weights of neighboring grids are set in advance. Similarly, GNN aggregates connected nodes to the central node. However, the influence of different nodes is different and always unknown, so the weights of different nodes should be adaptively adjusted. GAT is a special GNN model combined with an attention mechanism. As shown in Figure 3, the attention mechanism can aggregate the neighbor nodes of each node in the graph, and adaptively calculate the weights (α11, …, α15) of different neighboring nodes, representing the different importance degree. Compared with the advanced set weight in CNN and GNN, GAT can use the dynamic weights to improve the accuracy of the model [31].

The traditional prediction method uses the regular spatial grids as the carrier of traffic violations, ignoring the strong spatiotemporal relationship between traffic violations and road network. The spatial distribution of the road network is irregular, which is difficult to characterize by a CNN. However, GAT can better adapt to the irregular data structure. At the same time, the attention mechanism adjusts the influence weights of each node globally, which represent the interaction of research objects and different impact degrees on each other.

### 3.2. GATR

We propose a GATR (graph attention network based on road network) model. Considering the spatiotemporal correlation between the road network and traffic violations, the GATR model constructs a topological graph of them. We match the traffic violations on the graph, which can well express the spatiotemporal characteristics of traffic violations. Considering the significant impact of the urban function, GATR adopts urban functional features as an input feature, which is carried by road units.

**Figure 4:**
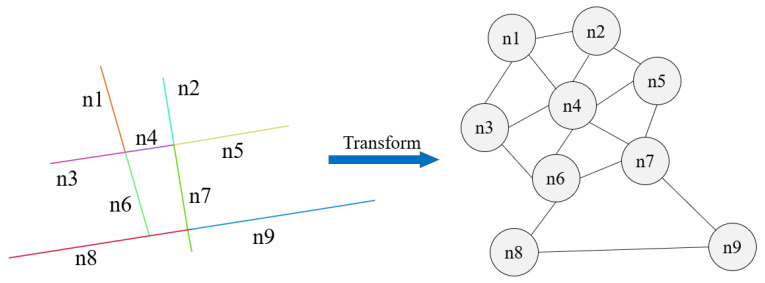
Graph representation of road network. Basic road units are the nodes of graph. Spatial reachability is the edges of graph. The number of traffic violations on the basic road unit is the predicted target value of every node.

The GATR model divides the road network into basic road units. They are taken as the nodes of the graph. Two road units whose distance is less than set threshold are regarded as connected and we will add an edge across them. The graph representation of road network is as shown in Figure 4. The nearest neighbor algorithm is used to spatially associate the traffic violation data with each basic road unit, and the number of traffic violations on the basic road unit is the predicted target value.

**Figure 5:**
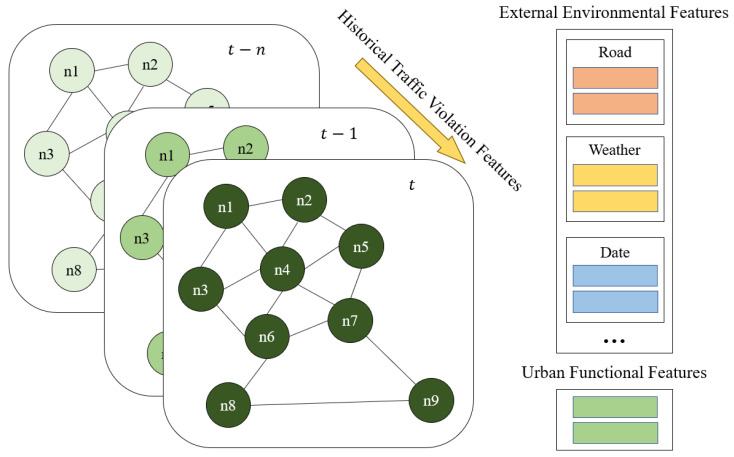
Feature set of traffic violations including historical traffic violation features, external environmental features and urban functional features.

We identify some features from multiple dimensions which have significant influence on the occurrence of traffic violations, which can be used as the input of GATR and help predict the spatiotemporal distribution of traffic violations. We consider the impact of historical traffic violation features combined the external environmental features and the urban functional features of roads, and these features make up the feature set. The feature set of the GATR model is as shown as Figure 5.

**Figure 6:**
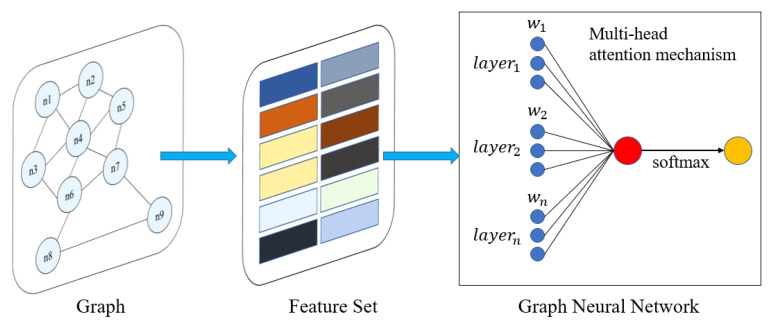
The architecture of GATR. The specific process is described as above.

The architecture of GATR is as shown in Figure 6. Firstly, taking the spatiotemporal correlation between traffic violations and the road network into account, the GATR model excavates the self and mutual influence between the basic road units and construct the topological graph. Secondly, GATR considers both the impact of the internal and external features of the traffic violations. These features determine the occurrence of traffic violations and can be used as input values of the GATR model. Finally, GATR predicts the spatiotemporal distribution combined with the attention mechanism. Compared with the traditional CNN model, the GATR model can better suppress the adverse the impact of spatiotemporal heterogeneity on the model performance and improve the prediction accuracy. At the same time, the irregular graph data structure of GATR model has a stronger spatiotemporal expression for traffic violations.

The detailed algorithm of the GATR model architecture is as follows:

1. Three matrices are generated, including graph matrix, feature matrix and label matrix. The ranks of these matrices are [N, N], [N, H, D], [N, 1, D]. N is the number of nodes. H is the number of the features. D is the number of days. The graph matrix records the connectivity between nodes. The feature matrix records the features of every node in every day. The label matrix records the traffic violation number of every node in every day.
2. In each training cycle, the data of different days is randomly selected for training. The feature data is input, the training result is compared with the label data, and the parameters are updated by backward propagation.
3. The selected data enters n attention layers and the results are spliced, which is the multiple attention mechanism. Then the spliced result enters into another attention layer and a Relu layer to complete a forward operation.
4. In an attention layer, first the data is entered into a linear layer to extract the features, then the data is calculated with its own transposition, then the data is filtered through the graph matrix. Finally, the data is entered into a Softmax layer and an offset is added to obtain the attention result.
5. After all training cycles are completed, a model for the spatiotemporal prediction of traffic violations is obtained, which is tested on the test data.

## 4. Experiment

### 4.1. Graph Construction Based on Road Network

The nodes of the topological graph are abstract research objects which are basic road units. There are 8550 roads in the original road network data. According to the guidance documents of relevant departments, the road network is divided into basic road units with a length of 500 m with a total number of 12,580. We take the basic road units as the nodes of the GATR model graph, set the roads within 1 km as the connection, and establish the edges of the nodes to build the graph of the GATR model. The first law of geography states that closer objects have higher correlation [32], and the spatial graph structure maintains the spatial adjacency through edges along the road network.

### 4.2. Feature Set

In order to explore the characteristics of traffic violations and improve the prediction accuracy, historical traffic violation features, external environmental features, and urban functional features are chosen as the feature set of traffic violations.

#### 4.2.1. Historical Traffic Violation Features

**Figure 7:**
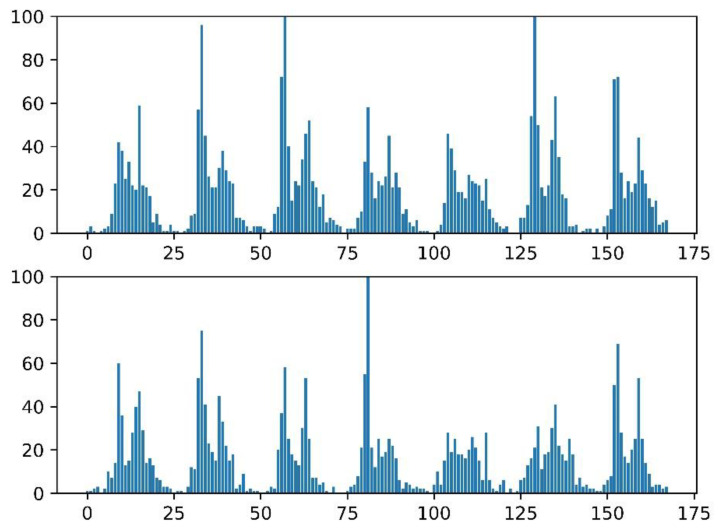
Cycle features of traffic violations by comparing the number of traffic violations per hour in two random weeks.

We randomly select two weeks from the dataset, and the numbers of traffic violations per hour are as shown in Figure 7. It can be found that traffic violations have significant periodic characteristics with a period of 7 days. Therefore, we take 7 days as the time series length of single prediction, in which the number of traffic violations in the first 6 days constitutes the features of historical traffic violations.

#### 4.2.2. External Environmental Features

External environmental features have an important impact on the traffic including relevant features of road network, temperature and weather, day of week, etc. [33,34] We quantitatively evaluate these external environmental features and include them into the feature set.

The road type and whether it contains an intersection of the basic road units are extracted as the road part of external environmental features. The road type attribute comes from OpenStreetMap, including 8 types of expressways, national highway, provincial highway, urban road class 1, urban road class 2, county highway, township village road, and others. The values of road type feature are as shown in Table 1. Some road units in the road network intersect with others. The intersection feature of the basic road units containing the intersection is assigned as 1, otherwise assigned as 0.

Temperature and weather are selected as the meteorological part of external environmental features. The temperature range is −10 °C to 40 °C. The weather description includes rainstorm, blizzard, heavy rain, heavy snow, moderate rain, moderate snow, thunderstorm, light rain, light snow, fog, haze, overcast, sunny, etc. According to the impact of weather on traffic conditions, the weather description is divided into four categories: extreme weather, worse weather, bad weather, and general weather. The corresponding feature values are shown in Table 2.

We select the day of week, season and whether it is a holiday as the typical date features of the basic road units.

#### 4.2.3. Urban Functional Feature

Urban function is the service provided by urban areas, such as commercial service, residential service, etc. Different city areas have different urban functions. Urban residents in different urban function areas need to achieve their unique goals in their daily life, so the functions of different areas around the city profoundly affect the traffic status [35,36,37]. A POI is a ground point with specific spatial location, name, category, and other information. The distribution of POIs in urban areas is closely related to urban functions. We can divide the city into functional areas based on the information of POI points and then explore the impact of urban functions on traffic violations.

TF-IDF algorithm is a statistical method which can evaluate the importance of words to individuals in corpus [38,39]. The study area is divided into spatial grids with a side length of 128. We measure the influence of various POIs by their number and global distribution in the grid [37,40]. The TF-IDF value is calculated as follows:

(1) $$TF_{x,y}=\frac{N_{x,y}}{N_{x}}$$

(2) $$IDF_{x}=\log\left(\frac{R}{R_{x}}\right)$$

(3) $$TF-IDF_{x,y}=TF_{x,y}\times IDF_{x}$$

$N_{x,y}$ is the number of POI points of type x in area y; $N_{x}$ is the total number of POI points of type x; R is the total number of areas; $R_{x}$ is the number of areas containing POI points of type x; $TF-IDF_{x,y}$ is the influence degree of POI points of type x on area y.

**Figure 8:**
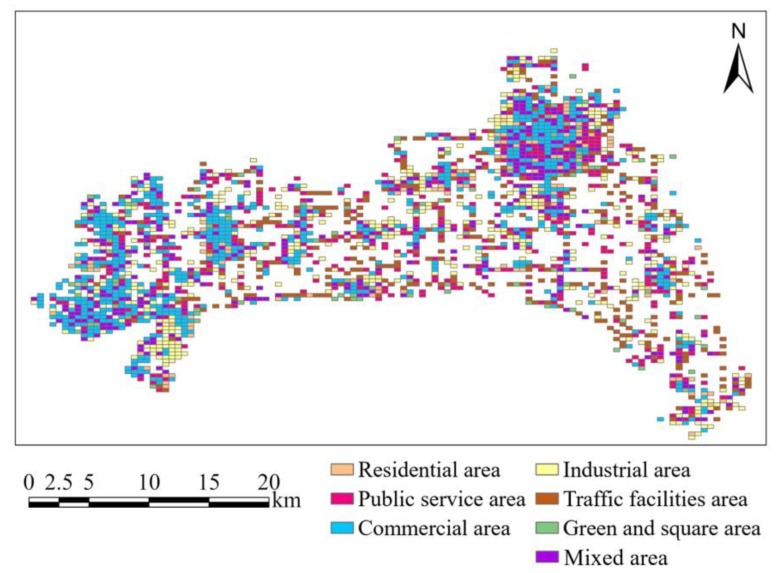
City H urban functional area distribution map.

**Figure 9:**
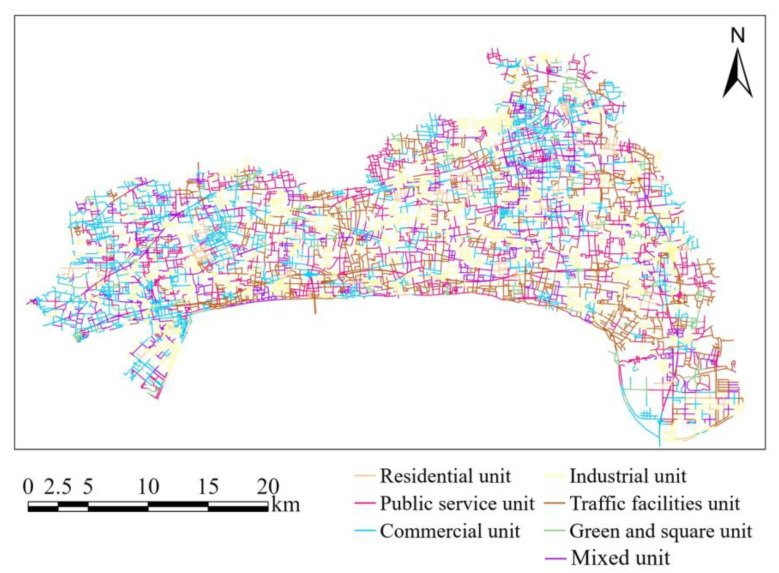
Urban functional features of basic road units.

The urban functions of the basic road units are input into the GATR model as urban functional features. We divided urban functional areas into 7 types, including residential area, public service area, commercial area, industrial area, traffic facilities area, green and square area, and mixed area. The division results are as shown in Figure 8. Then we utilize proximity analysis to assign values to the urban functional features of the basic road units. The urban functional features of the basic road units are as shown in Figure 9. Urban functional features of basic road units. The corresponding feature values are as shown in Table 3.

To sum up, we summarize 14 features of traffic violations on road units as the dataset of the GATR model, including 6 historical traffic violation features, 7 external environmental features, and 1 urban functional feature. Among them, the external environment features include 2 road features (road type, whether it is an intersection), 2 meteorological features (temperature, weather), and 3 date features (week, season, whether it is a holiday).

## 5. Results

### 5.1. Performance of GATR

We used the historical traffic violation dataset of City H as the experimental data. The time range was 1827 days from 1 January 2016 to 31 December 2020. The first 1747 days were used as the training dataset while the last 70 days (10 weeks) were used as the test dataset. The GATR model was trained and evaluated with the above summarized features as the input feature set of the graph. The number of traffic violations of each basic road unit was regarded as the tag value.

We chose RMSE (root mean squared error) as the index to evaluate the prediction accuracy, which can reflect the deviation between the predicted values and the true values. The value of RMSE is calculated as follows:

(4) $$RMSE=\sqrt{\frac{\sum_{i=1}^{N}{\left(P_{i}-A_{i}\right)}^{2}}{N}}$$

N is the number of nodes in the spatial graph; $P_{i}$ is the predicted traffic violation number on the node; $A_{i}$ is the actual traffic violation number on the node.

**Figure 10:**
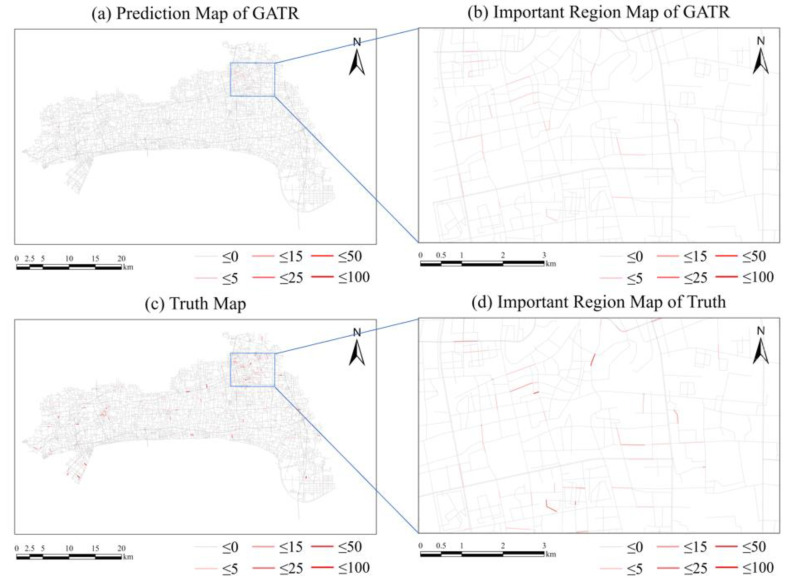
Comparison between the predicted result using GATR and the truth. (**a**) Predicted result of traffic violations using GATR. (**b**) Important region map of GATR. (**c**) Truth values of traffic violations. (**d**) Important region truth values of traffic violations.

**Figure 11:**
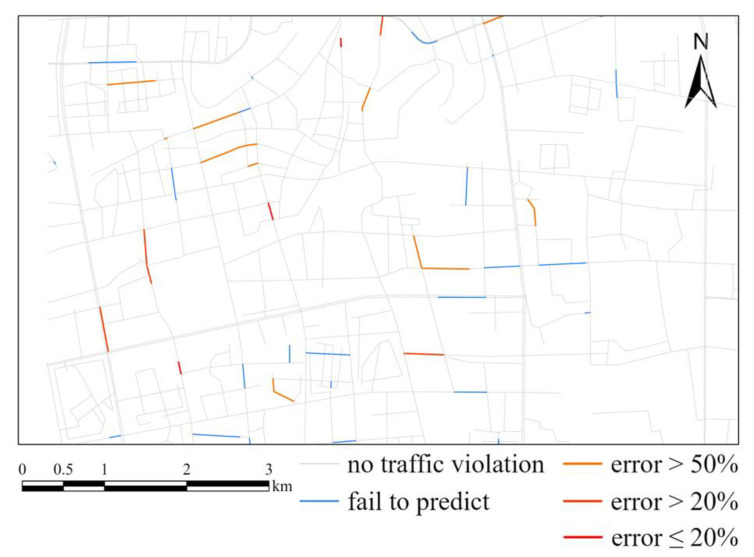
Comparison of GATR and truth. Blue lines represent roads having traffic violations but not predicted by GATR. Red lines represent well predicted roads.

Firstly, we used features except urban functional feature as the input. The input features include six historical traffic violation features and seven external environmental features. The RMSE of the test data after training was 1.7801. Taking 28 November 2020 as an example, the predicted value using the GATR model and the real value of traffic violations on that day are as shown in Figure 10, and the accuracy is shown as Figure 11, which show that the GATR model has a satisfactory performance of predicting the number and spatiotemporal distribution of traffic violations.

**Figure 12:**
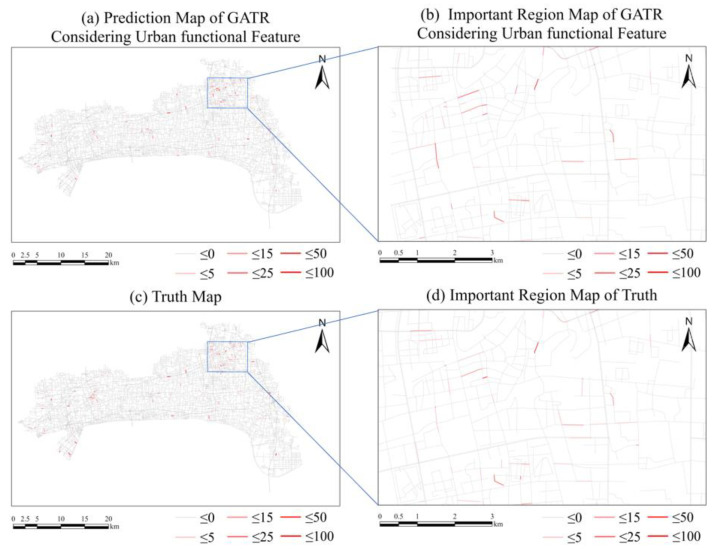
Comparison between the predicted result using GATR improved by urban functional feature and the truth. (**a**) Predicted result of traffic violations using GATR considering urban functional features. (**b**) Important region map of GATR. (**c**) Truth values of traffic violations. (**d**) Important region truth values of traffic violations.

**Figure 13:**
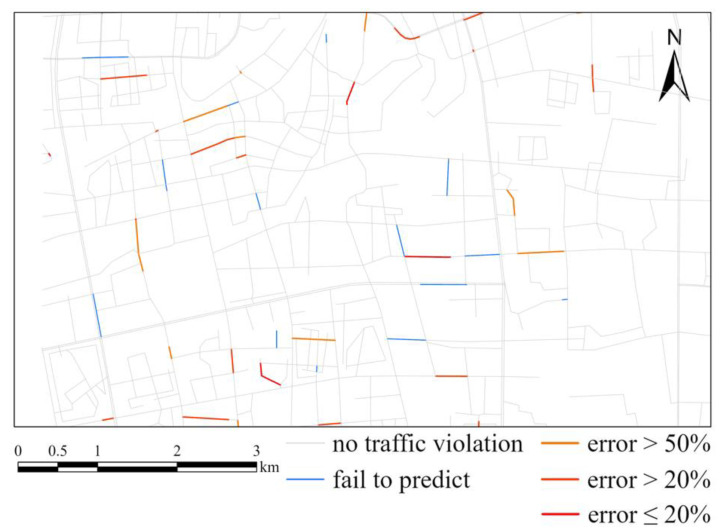
Comparison of GATR improved by urban functional feature and truth. Blue lines represent roads having traffic violations but not predicted by GATR. Red lines represent well predicted roads.

Based on the above experiments, we added urban functional features to optimize the GATR model. The RMSE of the test data was decreased to 1.7078. Similarly, taking 28 November 2020, in the test set as an example, the predicted value of GATR model and the real value of traffic violations on that day are as shown in Figure 12 and the accuracy is shown as Figure 13. It is obvious that more traffic violations can be predicted, and the accuracy was improved considering urban functional features. Furthermore, the spatial distribution pattern was greatly improved, which is more similar to the truth value. Thus, the optimized GATR model has better prediction ability for traffic violations.

### 5.2. Performance of Conv-LSTM

Existing traffic violation prediction methods include CNN, LSTM, Conv-LSTM, etc. CNN integrates adjacent regions around a target region through convolution, taking the spatial characteristics of traffic violations into account. LSTM extracts effective knowledge from a long time series, considering the time characteristics of traffic violations well. Conv-LSTM adopts the advantages of both CNN and LSTM, comprehensively considers the spatial and temporal characteristics of traffic violations, which achieves better prediction accuracy. Conv-LSTM is a widely used method for spatiotemporal prediction at present. Therefore, we selected Conv-LSTM as the comparison method.

**Figure 14:**
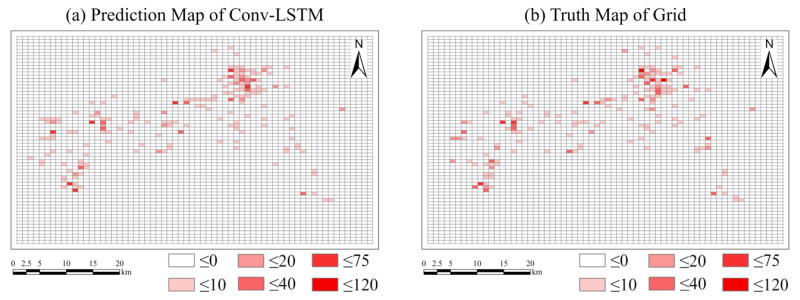
Comparison between the predicted result using Conv-LSTM and the truth. (**a**) Predicted result of traffic violations using Conv-LSTM. (**b**) Truth values of traffic violations divided by regular space grids.

The study area was divided into 64 × 64. The input data was also the traffic violation data of City H from 1 January 2016 to 31 December 2020. The RMSE of the test data after training was 2.1103. Similarly, taking 28 November 2020 as an example, the predicted value of the Conv-LSTM model and the real value of traffic violations on that day are as shown in Figure 14.

## 6. Discussions

### 6.1. Comparison between GATR and Conv-LSTM

In terms of the ability of spatial expression, GATR follows the rules of the road network and can locate traffic violations on the road units, while Conv-LSTM can only locate traffic violations on the grids. Compared with the regular grid division, the spatial distribution of basic road units is irregular, which can better reveal the spatial distribution law of traffic violations. The spatial expression is more refined.

**Figure 15:**
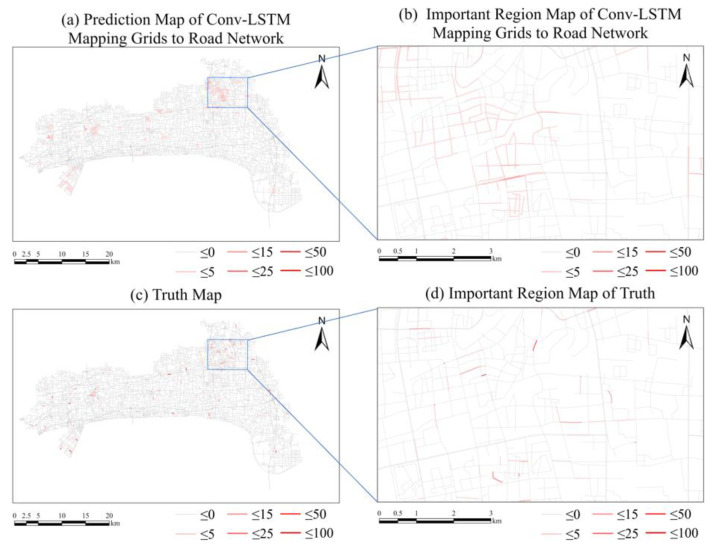
Comparison between the predicted result using Conv-LSTM (mapping the result of grids to the road network) and the truth. (**a**) Predicted result of traffic violations using Conv-LSTM (mapping the result of grids to the road network). (**b**) Important region map of Conv-LSTM. (**c**) Truth values of traffic violations. (**d**) Important region truth values of traffic violations.

In terms of prediction accuracy, the RMSE of GATR model is 1.7078, which is lower than that of the Conv-LSTM model (2.1103). In order to compare the prediction accuracy in the same dimension, we map the regular spatial grid result of Conv-LSTM to the road network by allocating the number of traffic violations in proportion to the length of each road in each grid. The mapped result of Conv-LSTM is as shown in Figure 15. We also recalculated the RMSE of the Conv-LSTM prediction in the road network, with a value of 1.9180, which is still higher than GATR, that is, GATR has higher prediction accuracy.

Through comparative experiments, we verify that the GATR model has stronger spatial expression and better prediction accuracy for traffic violations.

### 6.2. Interpretation of GATR Model Based on GNN Explainer

GNN Explainer can mine the subgraphs mostly related to the prediction results through the graph structure, and then form a certain interpretation ability for the prediction results of the GNN model. The graph mask and feature mask of GNN Explainer can filter and evaluate the nodes and features in the graph, respectively [41].

**Figure 16:**
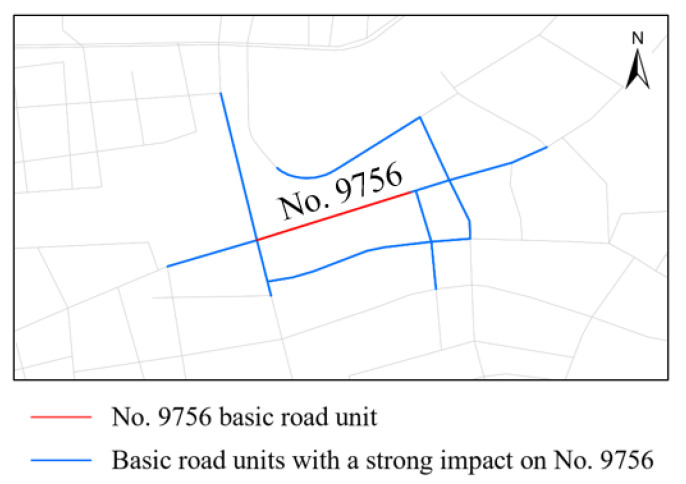
Road network influence subgraph with No. 9756 basic road unit as the core.

We use GNN Explainer to explain the GATR model. As shown in Figure 16, taking No. 9756 basic road unit on 28 November 2020 as the interpretation object, other basic road units that have a strong impact on it can be found. The subgraph of road network with No. 9756 as the core can be mined.

GNN Explainer can also explain the influence degree of input features in the prediction process of GATR model, which is as shown in Table 4. In the input feature set, the influence degree of weekday, urban functional, and historical traffic violation features is higher than 0.8, which has the most important affect. The influence degree of weather features is 0.788, which also has an important affect.

## 7. Conclusions

GATR model is proposed to model traffic violations and realize the spatiotemporal prediction of traffic violations. The GATR model associates traffic violations with the road network using basic road units to carry traffic violations and constructs a spatiotemporal graph. Compared with the Conv-LSTM, which is a classical model based on regular spatial grids, the GATR model has higher prediction accuracy and stronger spatial expression ability.

The input feature set of the GATR model not only considers the historical features of traffic violations, but also considers the external environmental features, including road features, meteorological features, and date features. Due to the strong correlation between traffic conditions and urban functions, we calculate the urban functional features of the area where traffic violations occur to improve the prediction accuracy of the GATR model. Experiments show that the GATR model has higher accuracy in predicting traffic violations than the existing CNN and LSTM methods.

Finally, we use GNN Explainer to explain the GATR model. The subgraphs of basic road units with strong influences around them can be mined to show the impact of each basic road unit on traffic violations. GNN explainer also explained the weight of each feature in the GATR model feature set, which quantitatively shows the impact of each feature on traffic violations.

In aspect of the features of traffic violations, GATR uses the urban function as a social feature, which reveals the unique impact of social features on traffic behavior. Social features are proved to be effective in traffic violation prediction, thus more social features should be considered in the further research. In the aspect of spatial expression of traffic violations, GATR creatively proposes a spatial graph of road network constraints to carry traffic violations. The spatial graph can not only more accurately express the spatial location of traffic violations, but also retain the spatial association of traffic violations through the road network. The spatial graph is a new research idea, instead of regular spatial grids for the traffic violation prediction.

With the help of GATR, we can more accurately predict the spatiotemporal distribution of traffic violations through various features. Because of the association between traffic violations and road networks, traffic violations can be accurately located, and then fixed-point prevention and control measures can be put forward for the road sections with high traffic violation risk. GATR can provide important reference for the improvement of traffic safety.

The topology of the graph used by GATR may be somewhat different from the original road network. We will further study and improve how to keep the topology consistent in the future. In addition, due to the limitations of data acquisition, GATR still has room for improvement in feature induction. More features that have an important impact on traffic violations can be considered for subsequent research.

## Figures and Tables

**Table 1:** The values of road type feature.

Road Type	Value
Expressway	7
National highway	6
Provincial highway	5
Urban road class 1	4
Urban road class 2	3
County highway	2
Township village road	1
Others	0

**Table 2:** The values of weather features.

Weather Description	Weather Categories	Value
Rainstorm, blizzard, heavy rain, heavy snow, etc.	Extreme weather	3
Moderate rain, moderate snow, etc.	Worse weather	2
Light rain, snow, fog, haze, etc.	Bad weather	1
Overcast, sunny, etc.	General weather	0

**Table 3:** The values of urban functional feature.

Urban Function	Value
Residential area	6
Public service area	5
Commercial area	4
Industrial area	3
Traffic facilities area	2
Green and square area	1
Mixed area	0

**Table 4:** The degree of input features.

Feature Name	Feature Categories	Influence Degree
Historical traffic violations	Historical traffic violation feature	0.828
Road type	External environmental features	0.469
Whether contains an intersection		0.551
Temperature		0.359
Weather		0.788
Weekday		0.903
Season		0.646
Whether it is a holiday		0.698
Urban functional feature	Urban functional feature	0.847

## Data Availability

The traffic violation dataset is obtained from Key Laboratory of Public Security Informatization Application Based on Big Data Architecture, China, which is not publicly available due to privacy and confidentiality restrictions. Other datasets in this study are publicly available, which can be found here: “https://www.openhistoricalmap.org/”, “http://lishi.tianqi.com/”, https://wannianrili.bmcx.com/” and “https://lbs.amap.com/”.
